# The influence of environmental enrichment and stocking density on the plumage and health conditions of laying hen pullets

**DOI:** 10.3382/ps/pez024

**Published:** 2019-02-01

**Authors:** Christopher J Liebers, Angela Schwarzer, Michael Erhard, Paul Schmidt, Helen Louton

**Affiliations:** 1 Department of Veterinary Sciences, Chair of Animal Welfare, Ethology, Animal Hygiene and Animal Husbandry, Faculty of Veterinary Medicine, LMU Munich, Veterinaerstrasse 13/R, 80539 Munich, Germany; 2 Statistical Consulting for Science and Research, Zimmerstr. 10, 76327 Pfinztal, Germany

**Keywords:** rearing period, pullet, environmental enrichment, stocking density, plumage condition

## Abstract

In this study, the effects of environmental enrichment, stocking density, and microclimate on feather condition, skin injuries, and other health parameters were investigated. During 2 rearing periods (RP), non-beak-trimmed Lohmann Brown hybrid pullets were housed in an aviary system for rearing with cages and from week 5 of age onwards with access to a litter area. All pullets were reared in the same barn and under practical conditions. In total, 9,187 (RP 1) and 9,090 (RP 2) pullets were distributed in 9 units, and each unit was assigned to 1 of 3 experimental groups (EG). In the control group (EG 1), the pullets were kept without environmental enrichment and at a commonly used stocking density (22 to 23 pullets per m²). Each unit of the 2 treatment groups was provided with 3 types of environmental enrichment simultaneously (pecking stones, pecking blocks, and lucerne bales), and the pullets were kept at a lower than usual (18 pullets per m²) (EG 2) or commonly used stocking density (EG 3). In each RP, the plumage condition, injuries and health of the pullets, and the microclimate of the housing system were examined 5 times. The statistical relationships of enrichment, stocking density, and microclimate with animal health were estimated via regression models. We found that the provision of environmental enrichment had a significant increasing effect on the plumage quality in week 17. Furthermore, significant relationships were found between several predictors (temperature in the housing system, dust concentration, and age of the pullets) and response variables (plumage condition, body injuries, head injuries, bodyweight, difference to the target weight and uniformity). The results of this study showed that increasing temperature in the housing system and increasing age of the pullets are significantly associated with the occurrence of feather damage and skin injuries during rearing. With stocking densities as high as we used (all > 17 pullets per m²), no significant positive effect of a reduced stocking density could be observed.

## INTRODUCTION

The practice of beak trimming does not solve the underlying problems of feather pecking and cannibalism in laying hens, although it drastically decreases general plumage damage (Lee and Craig, 1991; Sun et al., 2014; Hartcher et al., 2015) and mortality due to cannibalism (Guesdon et al., 2006; Sun et al., 2013, 2014). With the abandonment of beak trimming as already realized in several European countries, e.g., in Norway, Sweden, Austria, and Germany, new strategies have to be found and implemented to prevent feather pecking and cannibalism and to ensure animal welfare. The rearing period (**RP**) plays an important role in the prevention of the before-mentioned unwanted behavior (Hoffmeyer, 1969; Blokhuis and Arkes, 1984; Vestergaard and Lisborg, 1993; Vestergaard et al., 1993; Sanotra et al., 1995; Vestergaard and Baranyiová, 1996; Johnsen et al., 1998; Gunnarsson, 1999; McAdie et al., 2005; Bestman et al., 2009; Lambton et al., 2010).

Feather pecking is a problem in all types of housing systems, and it occurs during rearing as well as during the laying period (Wells, 1972; Hansen and Braastad, 1994; Huber-Eicher and Audigé, 1999; Sarica et al., 2008; Bestman et al., 2009; Gilani et al., 2013; Tahamtani et al., 2016; Widowski et al., 2017). Feather pecking describes the act of pecking and pulling out feathers from another bird. This behavior results in plumage damage and potentially in skin injuries (Bilcik and Keeling, 1999). The plucking of feathers is probably painful for the victim and therefore affects animal welfare (Gentle and Hunter, 1991). Furthermore, feather pecking is of economic interest because thinner feather coverage leads to a higher food intake to compensate the increased heat loss (Leeson and Morrison, 1978; Damme and Pirchner, 1984). Despite the damage it causes, feather pecking clearly differs from aggressive pecking behavior (Hoffmeyer, 1969). It is assumed to be the result of misdirected foraging behavior due to a lack of appropriate foraging material or missing experience with sufficient dust bathing substrate (Hoffmeyer, 1969; Blokhuis and Arkes, 1984; Vestergaard and Lisborg, 1993; Vestergaard et al., 1993). Young chickens learn about pecking and dust bathing substrate during their first days of life (Sanotra et al., 1995; Vestergaard and Baranyiová, 1996). In contrast to feather pecking, aggressive pecking is motivated by dominance and the forming of a social hierarchy, and the pecks are usually directed at the head (Savory, 1995). Cannibalism describes the pecking of skin and underlying tissue of conspecifics (Keeling, 1994). This behavior can be divided into pecking of denuded skin areas or vent pecking (Savory, 1995). The pecking of exposed skin areas increases even more after the appearance of hemorrhages. Vent pecking occurs especially at the beginning of lay and at the moment when the cloacal mucous membrane is prolapsed after the hen laid an egg. Cannibalism is a major problem in terms of animal welfare, and it leads to economic losses due to increased mortality.

The German Order on the Protection of Animals and the Keeping of Production Animals (2006) demands that laying hens should be reared according to their later housing conditions. In addition, it is beneficial to provide laying hen pullets with suitable litter, mash food, and environmental enrichment. The stimulation of foraging behavior with sufficient substrate keeps the pullets from pecking at each other during the RP (Huber-Eicher and Wechsler, 1998; Gilani et al., 2013), and it is important to prevent feather pecking in this early stage of life because it is hard to stop once it occurred (Bestman et al., 2009).

The reduction of stocking density is another factor that is reported to have various beneficial effects on animal welfare (Wells, 1972; Ali and Cheng, 1985; Carey, 1987; Cunningham and Gvaryahu, 1987; Davami et al., 1987; Hansen and Braastad, 1994; Tauson and Abrahamsson, 1994; Huber-Eicher and Audigé, 1999; Nicol et al., 1999; Onbaşılar and Aksoy, 2005; Sarica et al., 2008; Bestman et al., 2009; Widowski et al., 2017). Nonetheless, there are no legal requirements in Germany and the European Union regarding the rearing conditions, including stocking density, for laying hen pullets in commercial aviary systems. However, the Lower Saxony State Office of Consumer Protection and Food Safety (2013) published a guideline with recommendations on the rearing of laying hen pullets, recommending a stocking density of 18 pullets per m², as used in our study.

In this study, we examined and analyzed the effects of environmental enrichment, stocking density, and microclimate on the welfare of non-beak-trimmed laying hen pullets in order to compare the conventional rearing system in Germany with alternatives. The aim of this project was to find and investigate new solutions that effectively prevent feather pecking and health problems during the RP.

## ANIMALS, MATERIALS AND METHODS

### Animals and Housing System

The study was conducted on a conventional pullet rearing farm in Germany. The barn that we used had a rearing capacity for 100.000 pullets. In total, 9,187 (RP 1) and 9,090 (RP 2) pullets participated in our study and were reared under practical conditions in this barn. Assessments were made during 2 successive RP in 3 experimental groups (**EG**) with 3 units each. While RP 1 took place from July to October, RP 2 took place from December to April. All pullets were non-beak-trimmed Lohmann Brown hybrids of the same age. Transportation of the 1-day-old pullets was carried out by LSL Rhein-Main, Dieburg, Germany. Each RP lasted approximately 18 wk. Subsequent to the RP, the pullets were sold to conventional egg producers. In both RP, the pullets were vaccinated against Marek's disease, coccidiosis, infectious bronchitis, Salmonella, Newcastle disease, infectious bursal disease, infectious laryngotracheitis, avian encephalomyelitis, and *Escherichia coli* (only RP 1).

The barn was equipped with the aviary rearing system Meller Type 501-3 (Meller International GmbH, 49324 Melle, Germany). During the winter months, the ventilation of the barn was achieved by 5 ventilators (Ziehl Abegg FC 91, Ziehl-Abegg SE, 74653 Künzelsau, Germany) placed in the middle of the barn. Fresh air came into the barn via vents in the roof area. During the summer months, a tunnel ventilation system was used additionally, with 8 ventilators at one end of the barn and vents in the roof at the other end of the barn. The ventilation system could move 4.8 m³ of air per pullet per hour. Heating was supplied by a gas heating system which consisted of gas guns. Four gas guns were positioned next to the 9 units we observed. The aviary system consisted of several aviary segments (length: 2.41 m each) with 3 cage levels and a litter area. Four (units 1 to 3) or 5 (units 4 to 9) aviary segments were defined as 1 of 9 units. The number of animals in each unit and segment can be seen in Table 1. The units were positioned in a row on the left side of the barn. Unit 1 was in front and nearest to the ventilation fans, unit 9 was in the back of the barn. Each EG had a unit in the front, middle, and at the end of the row in order to compensate different effects of the position in the barn. The units were separated by metal plates between the aviary segments and the litter areas were separated by closed mesh wire doors. The whole cage row was separated from the next cage row (which was not part of the study) by mesh wire. The middle and lower cage levels in every aviary segment had a food conveyer belt, water supply from 8 nipple drinkers, and 2 round metal perches each. The top level had 8 nipple drinkers and 6 round metal perches at different heights. The nipple drinkers were lifted to accommodate the growing pullets. The food conveyer belts and perches reached through the whole length of the aviary segment. Each cage level had wired mesh (grid size 17 × 36 mm) on the bottom and a manure conveyer belt beneath.

**Table 1.** Distribution of the pullets, stocking densities, and the enrichment in the 9 units.

EG	1	2	3
Unit	3, 5, 8	1, 6, 7	2, 4, 9
Enrichment	No	Yes	Yes
P./m² ground surface	38.48	34.00	38.48
Pullets per unit			
Planned	920 (Unit 3) 1150 (Units 5 and 8)	812 (Unit 1) 1015 (Units 6 and 7)	920 (Unit 2) 1150 (Units 4 and 9)
Actual (RP 1, RP 2)	926, 880 (Unit 3) 1131, 1115 (Units 5 and 8)	823, 825 (Unit 1) 1034, 1019 (Units 6 and 7)	893, 903 (Unit 2) 1108, 1111 (Units 4 and 9)
Pullets per aviary segment			
Planned	230	203	230
Actual (RP 1, RP 2)	228, 222	206, 205	222, 223
Usable area on day 10, p./m²			
Planned	119.3	105.3	119.3
Actual (RP 1, RP 2)	118.3, 114.9	107.1, 106.1	115.2, 115.8
Usable area on day 35, p./m²			
Planned	22.8	18.0	22.8
Actual (RP 1, RP 2)	22.6, 22.0	18.3, 18.2	22.1, 22.2
Usable litter area on day 50, p./m²			
Planned	80.9	50.1	80.9
Actual (RP 1, RP 2)	80.2, 77.9	51.0, 50.6	78.1, 78.5

The actual numbers of pullets in the units within each experimental group (EG) differed slightly from each other and from the planned numbers. The mean values from the 3 units in each EG are presented in the table.

P./m² = pullets per m², RP = rearing period.

The pullets were distributed into the 9 units so that 3 units always had the same stocking density, were provided with environmental enrichment or not, and were defined as 1 of 3 EG, as seen in Table 1. The RP had a cage phase (day 1 to week 5 of age) and an aviary phase (week 5 to week 18 of age). The 1-day-old pullets were all placed in the middle cage level of each aviary segment; at 10 D of age, half of the pullets were placed in the lower cage level to meet the growing need for more space. During the cage phase, the pullets could not move between the cage levels and had no access to the litter area. During the first weeks, the mesh wire was covered with a layer of paper over which chicken feed was spread. During RP 1, the paper was partially removed at 15 D of age due to mold formation and completely removed at 29 D of age. During RP 2, the paper was completely removed at 15 D of age in order to prevent the formation of mold in the first place. At the beginning of the aviary phase, the cage levels were opened by folding down the doors to now function as “balconies,” giving the pullets access to the litter area and the other levels. A conventional feeding program was used with deuka feed from Deutsche Tiernahrung Cremer GmbH & Co. KG (93055 Regensburg, Germany): “Kükenstarter gekörnt” (chicken starter granulated) for the first 12 D, “All-mash A” (mash) from week 3 to week 8 and “All-mash R” (mash) from week 9 to week 18. The animals were fed ad libitum, and the feeder space per pullet was 2.1 cm (EG 1), 2.3 cm (EG 2), and 2.2 cm (EG 3).

### Environmental Enrichment and Litter

The enrichment consisted of pecking stones, pecking blocks, and lucerne bales. The pecking stones (VILOLith PICKStein Geflügel, Deutsche Vilomix Tierernährung GmbH, Neuenkirchen-Vörden, Germany) were based on mineral components, containing calcium, magnesium, sodium, and trace elements, and weighed 8 to 10 kg. The pecking blocks (PICKBLOCK, Crystalyx Products GmbH, Münster, Germany) consisted of selected cereals, minerals, and fiber components, had a structure of fine and rough constituents and weighed 5 kg. If they were used up completely (100%), the stones and blocks were replaced in the cage levels during the cage phase and in the litter area for the remaining 13 wk. The lucerne bales (Hartog Compact Luzerne, Grasdrogerij Hartog BH, Lambertschaag, Netherlands) consisted of dried and heated lucerne, compressed into 20 kg bales, and kept in form with plastic straps. The lucerne bales were replaced 1 to 2 times in each RP.

During the first days of life, all pullets in 1 segment (Table 1) shared one-sixth (1.3 to 1.7 kg) of a pecking stone and one-half (2.5 kg) of a pecking block in the middle cage level simultaneously. At 10 D of age, half of the pullets were placed in the lower cage level and both cage levels were provided with the same amount of enrichment devices as before. When the cage levels were opened and the pullets were given access to the litter area and the entire unit, all pullets of the unit had access to 1 whole pecking stone and block (in RP 2, 2 whole blocks at the same time) in the litter space and 1 lucerne bale simultaneously.

The litter area of all units was covered with a layer of long-cut straw.

### Methods of Assessment

Data were collected over 2 RP.

#### Assessment of Animal Health.

During each RP, the pullets were assessed 5 times (A1 to A5) as follows: A1 = 3rd week of age: day 19 (RP 1) or day 17 (RP 2); A2 = 5th week of age: day 32 (RP 1) or day 31 (RP 2); A3 = 8th week of age: day 54 (both RP); A4 = 12th week of age: day 81 (RP 1) or 13th week of age: day 87 (RP 2); A5 = 17th week of age: day 116 (RP 1) or day 115 (RP 2). At every visit, 50 pullets were assessed randomly from each unit. In total, 4,500 pullets were examined. The pullets were weighed on a scale (Weighing Terminal Type: ICS425s, Mettler-Toledo GmbH, Albstadt, Germany) and then examined in detail with a modified pullet score system (Gunnarsson, 2000; Tauson et al., 2005) (Table 2). The examination of the pullets was divided into the following regions: head and body. For the evaluation of the plumage condition, a “triscore” was used comprising the plumage score data of dorsal neck, back, and left wing. The lowest possible score was 3 and the highest was 12. A score of 12 describes a full plumage, whereas a score of 3 represents a pullet with bald patches in all mentioned body regions. In the assessment of the plumage quality, we attempted to include only pecking damage but not feather damage due to molt. The assessment of the body injuries was conducted in 9 body regions and categorized into ≤0.5 cm and >0.5 cm as seen in Table 2. The variable “head injuries” includes general head injuries and injuries of comb and eyelids. The mean bodyweight, the difference to the target weight, and the uniformity were calculated for each unit. The difference to the target weight describes the missing or surplus percentage of the target weight. The target weight we used followed the recommendations by Lohmann Tierzucht GmbH (2017).

**Table 2.** Assessment of pullet health and the scores given.

Parameter	Alteration	Score
	None	4
	≤5 feathers affected	3
Damaged feathers^1^		
	>5 feathers affected	2
	Bald patches <1 cm	1
	None	0
Body injuries^2^	≤0.5 cm	1
	>0.5 cm	2
	No	0
Head injuries^3^		
	Yes	1

^1^Damaged feather: missing feathers, broken or interrupted parts. Feathers were assessed on dorsal neck, back, and left wing.

^2^Body injuries were assessed on dorsal and ventral neck, breast, abdomen, back, left wing, left thigh, tail, and cloaca.

^3^Head injuries included injuries on the head, eyelids, and comb.

The assessment followed a modified pullet score system according to Gunnarsson (2000) and Tauson et al. (2005).

#### Assessment of Environmental Enrichment and Litter.

The stones and blocks were weighed on a scale (Page Profi, Soehnle, Nassau, Germany), and the weight was recorded at each assessment time (A1 to A5). The quality of the litter in the litter area was examined, evaluated and recorded during assessments A3 to A5. During assessments A1 and A2, the pullets did not have access to the litter area. The evaluation of the litter was conducted in 1 location, always in the middle of the litter area of each unit. The evaluation score used is based on the Welfare Quality assessment protocol for poultry (Welfare Quality®, 2009): 0 = completely dry and flaky, i.e., moves easily with the foot; 1 = dry but not easy to move with the foot; 2 = leaves imprint of foot and will form a ball if compacted, but does not hold its form; 3 = sticks to boots and sticks readily in a ball if compacted. Prior to the assessment of quality, the litter depth was measured.

#### Microclimate.

During each assessment (A1 to A5), the microclimate was examined. Light intensity, ammonia concentration, temperature, humidity, and dust concentration were measured. All of the data were recorded at the height of the heads of the pullets (standing upright) in the middle of the litter area of each unit. Except for the values for light intensity, which were taken in the middle cage level of each unit. Light intensity was assessed with an LMT Pocket-Lux 2B (LMT Lichtmesstechnik GmbH, Berlin, Germany) using a 6-point measuring system. Ammonia was documented using 2 MSA NH3 Altair Pro (MS Auer, Berlin, Germany). Temperature was assessed using a Testo 925 (Testo AG, Lenzkirch, Germany), and humidity was measured with a Testo 410-2 (Testo AG, Lenzkirch, Germany). The dust concentration was recorded with a Dust Trak DRX Aerosol Monitor TSI (TSI, Inc., 500 Cardigan Road, Shareview, MA). We tried to make sure that the measurements for the dust concentration were taken at a similar time period each assessment visit and that before measuring no management arrangements were conducted that would increase the dust concentration. The measured dust concentrations were divided into 3 categories according to particle size: particulate matter 10 (PM10, <10 μm), particulate matter 2.5 (PM2.5, <2.5 μm), and total concentration (particles <100 μm). Additionally, the outside temperature was taken from the closest official German weather station in Regensburg, Germany, for a period of 2 wk before each assessment date, in order to evaluate the average outside temperature.

### Statistics

All analyses were carried out using the statistical programming language R (R Core Team, 2017). For the statistical analysis, the relationships between the predictors (environmental enrichment, stocking density, light intensity, housing temperature, dust concentration, age of the pullets) and the response variables (plumage condition, body injuries, head injuries, bodyweight, difference to the target weight and uniformity) were analyzed simultaneously on the basis of the units. In addition to the above-listed predictors, which were modeled as fixed effects, the units were included as unstructured random effects. As a result, the models used are linear mixed models. All response variables are continuous variables and for the observational model a normal distribution has been chosen. The resulting generalized mixed models were fitted using the lme4 package for R. Missing data in the dataset were imputed multiple (50) times by using the Amelia package (Honaker et al., 2011).

**Figure 1.**
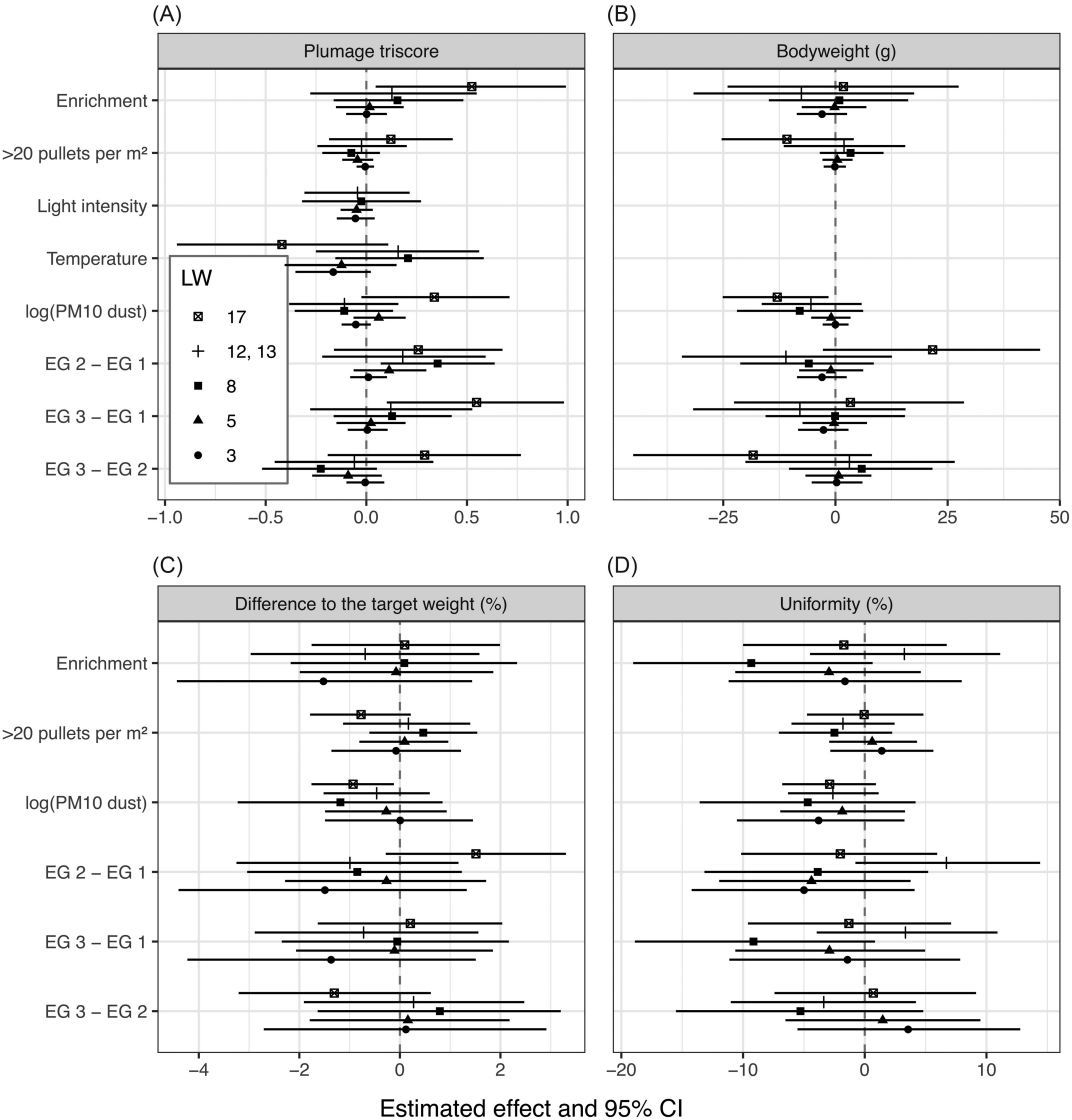
Estimated effect of predictors on the response variables in the different weeks of life. Estimated effects (solid symbols) and 95% confidence intervals (CI; bars) are shown in the diagram for plumage triscore (A), bodyweight (B), difference to the target weight (C), and uniformity (D). If all values of the CI are either positive or negative, the effect is considered significantly increasing or decreasing, respectively. The wider the CI, the less precise is the estimation. The size of the effect can be seen in the distance of the estimated effect from the zero line. However, the scales of the diagrams are standardized and the actual size of the estimated effect can only be seen in the text for the significant effects. For each estimation n = 18 was used in the statistical analysis. EG = experimental group, PM10 = particulate matter 10 (particles < 10 μm).

**Figure 2.**
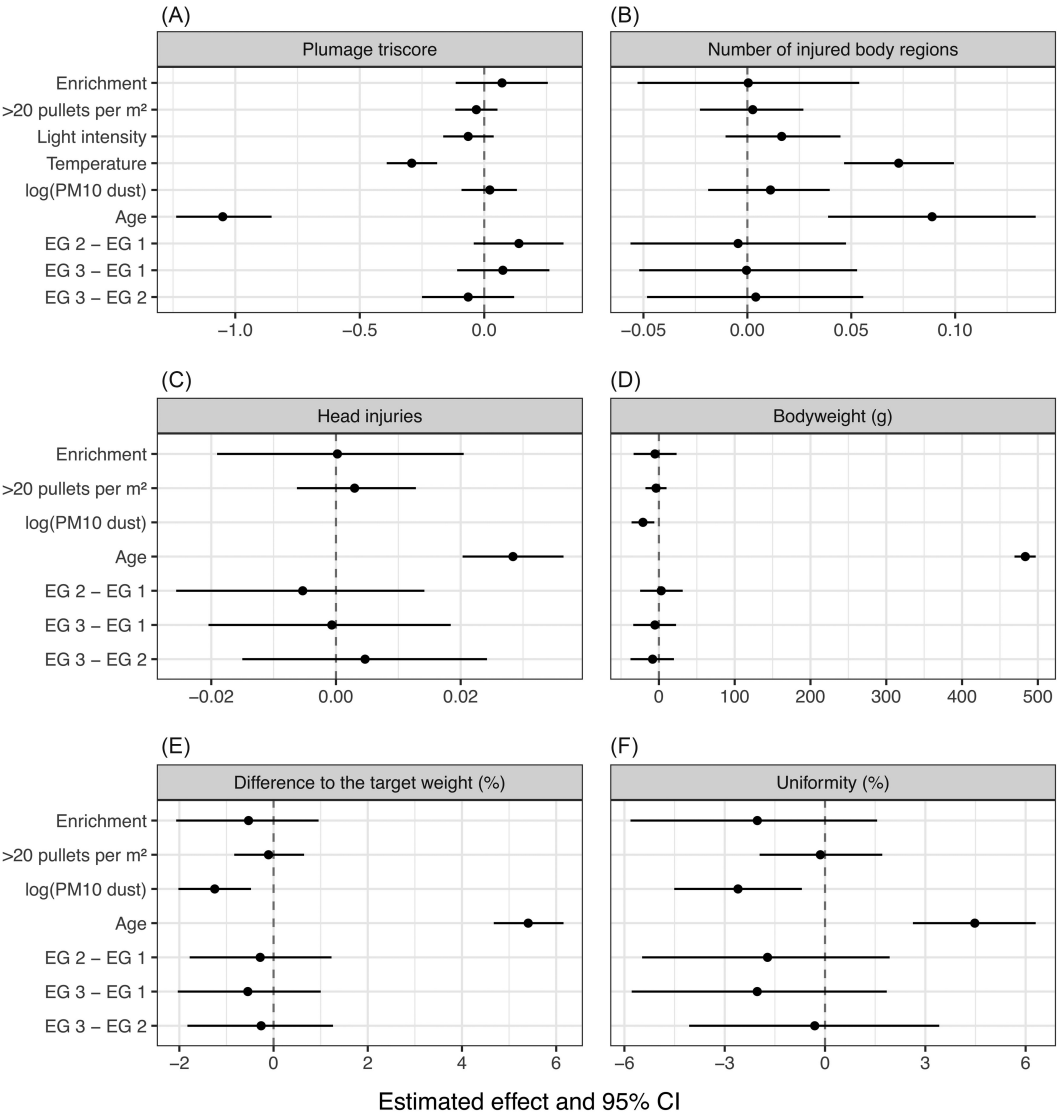
Estimated effect of predictors on the response variables. Estimated effects (solid circles) and 95% confidence intervals (CI; bars) are shown in the diagram for plumage triscore (A), the number of injured body regions (B), head injuries (C), bodyweight (D), difference to the target weight (E), and uniformity (F). If all values of the CI are either positive or negative, the effect is considered significantly increasing or decreasing, respectively. The wider the CI, the less precise is the estimation. The size of the effect can be seen in the distance of the estimated effect from the zero line. However, the scales of the diagrams are standardized and the actual size of the estimated effect can only be seen in the text for the significant effects. For each estimation n = 90 was used in the statistical analysis. EG = experimental group, PM10 = particulate matter 10 (particles < 10 μm).

Following the recent ASA’s statement on *P* values (Wasserstein und Lazar, 2016) and the discussion therein (McShane and Gal, 2017), we decided to forgo the use of *P* values. Instead we followed an approach that emphasizes estimation over testing, i.e., where hypotheses are identified by parameters within a specific regression model. In this approach statements about hypotheses of interest are based solely on the direction of an effect, its size and its precision (Figures 1 and 2). Results are statistically significant when the precision of an estimate is so high that the effect points in a clear direction (the 95% confidence interval [**CI**] does not cross zero). In this paper, the size of an effect is measured by its point estimate (the further away from zero, the greater the size), its direction is given by the estimated sign (a negative point estimate represents a decreasing effect, a positive point estimates an increasing effect), and its precision is represented by 95% CI (the wider the 95% CI, the less precise the estimation). The actual size of the point estimate cannot be seen in the figures because the scales of the graphics are standardized. The actual size can only be seen for the significant effects with the help of regression coefficients in the text.

Ammonia causes difficulties in the estimation of its effects on the response variables because 51.1% of all data have a value of 0, and 20% of the data are missing due to a defect measuring instrument. In addition, there are simple (in RP 1, all measurements had a value of 0) and complex relationships with other predictors. All this combined made it impossible to estimate the effect of ammonia on the response variables. The distribution of the predictor dust concentration was highly skewed, which justified an analysis on the logarithmic scale.

## RESULTS

### Animal Health

The plumage triscore is the sum of the plumage score data of the back, neck, and wing. The score ranges from 3 to 12 with 3 being the lowest and 12 being the highest possible score. In the study, no score value under 6 was found. The average value of the plumage triscore was similar in all 3 EG as shown in Table 3. The provision of environmental enrichment had a significant increasing effect on the plumage condition in week 17 (regression coefficient: 0.52; 95% CI for the difference: 0.05 to 0.99) and led to a significantly better triscore in EG 3 (9.45) compared to EG 1 (9.04) (regression coefficient: 0.55; 95% CI for the difference: 0.10 to 0.98) (Figure 1A). The reduction of the stocking density did not have a significant effect in any week or throughout the whole RP (Figure 2A). In week 8, EG 2 showed a significantly higher triscore (10.66) than EG 1 (10.34) (regression coefficient: 0.35; 95% CI for the difference: 0.07 to 0.64) (Figure 1A). In general, EG 2 had a slightly higher plumage triscore (on average 10.61) than EG 1 (on average 10.40) (Table 3). However, this difference was not statistically significant (Figure 2A).The plumage quality decreased significantly with increasing temperature in the housing system (regression coefficient: –0.06; 95% CI for the difference: –0.08 to –0.04) and with increasing age of the pullets (regression coefficient: –0.21; 95% CI for the difference: –0.25 to –0.17). The development of the triscore was similar in all 3 EG during both RP but differed between RP 1 and RP 2. At an age of 3 wk, the plumage condition had a mean score of 11.88, and in week 17 it was on average 9.31. In RP 2, the plumage quality decreased relatively evenly, whereas the decrease in RP 1 proceeded first slowly but then accelerated from A2 to A3 and from A3 to A4. During this time, the decrease of the triscore from 1 assessment visit to the next was on average >1 in all 3 EG. Towards the end of RP 1, the further decrease of the plumage condition was only minor in EG 1, whereas a slight improvement in the plumage quality was found in EG 2 and EG 3. During RP 1, the plumage triscore was in general lower than in RP 2.

**Table 3.** The results for plumage triscore, number of injured body regions, and head injuries in each experimental group (EG) throughout all weeks of life.

Response	EG	EE	P./m²	n	Mean	SD	Min.	Max.
	1	no	>20	30	10.40	1.12	8.44	11.98
Plumage triscore	2	yes	<20	30	10.61	1.03	8.62	12.00
	3	yes	>20	30	10.55	1.05	8.74	12.00
	1	no	>20	30	0.13	0.11	0.00	0.36
Number of injured body regions	2	yes	<20	30	0.11	0.13	0.00	0.66
	3	yes	>20	30	0.11	0.11	0.00	0.44
	1	no	>20	30	0.02	0.05	0.00	0.22
Head injuries	2	yes	<20	30	0.02	0.03	0.00	0.10
	3	yes	>20	30	0.02	0.06	0.00	0.30

None of these differences were significant.

EE = environmental enrichment, P/m² = pullets per m², n = number of units included in the calculation, SD = standard deviation, Min. = smallest assessed value, Max. = largest assessed value.

Body injuries were located most commonly on the tail (4.4%), the back (3.7%), and the cloaca (3.1%) (Table 4). Other body regions were rarely affected. Most of the injuries (91%) had a size of ≤0.5 cm. In the following, the exact location of the body injuries was considered irrelevant and the focus was on the number of injured regions on each animal (Table 3). In general, 89.5% of all animals in all groups had no injuries, 9.6% had 1 affected body region, 0.9% had 2, and 0.1% had 3. In both EG with environmental enrichment, the pullets showed slightly fewer injured body regions (0.11) than the pullets in the EG without enrichment (0.13). However, this difference was not statistically significant (Figure 2B). The stocking density did not seem to have an effect on body injuries. The number of affected body regions increased significantly with increasing temperature in the housing system (regression coefficient: 0.02; 95% CI for the difference: 0.01 to 0.02) and with increasing age of the pullets (regression coefficient: 0.02; 95% CI for the difference: 0.01 to 0.03) (Figure 2B). At the age of 5 to 8 wk, the pullets showed injuries at a progressive rate (on average 0.13 injured body regions per pullet). Most of the injuries were recorded around week 8 in RP 2 (0.16 injuries) and week 12 in RP 1 (0.34 injuries). In the following weeks, the level of injuries declined. Towards the end of the RP, the pullets had an average of 0.18 (RP 1) and 0.09 (RP 2) injured body regions. When comparing RP 1 with RP 2, the pullets in RP 1 showed in general a slightly higher level of injuries (0.13 injuries) than those in RP 2 (0.10 injuries).

**Table 4.** Distribution and number of injuries of the 9 assessed body regions in descending order, beginning with the most affected body region as an average of all experimental groups.

Body region	No skin injuries (%)	Injuries ≤0.5 cm (%)	Injuries >0.5 cm (%)
Tail	95.58	4.09	0.31
Back	96.29	2.98	0.73
Cloaca	96.93	3.02	0.04
Neck, ventral	99.84	0.13	0.02
Thigh	99.87	0.13	0.00
Abdomen	99.96	0.04	0.00
Neck, dorsal	99.98	0.02	0.00
Wing	99.98	0.02	0.00
Breast	100.00	0.00	0.00

Because none of the assessed pullets showed head injuries in all 3 assessed regions of the head at the same time, we analyzed whether any kind of head injury was recorded. Of all pullets, 1.9% had any kind of head injury. The number of head injuries per pullet was the same in each EG and was not influenced by enrichment or stocking density (Table 3). However, its occurrence was positively correlated with increasing age of the pullets (regression coefficient: 0.006; 95% CI for the difference: 0.004 to 0.007) (Figure 2C).

When comparing the 3 EG with each other, the mean bodyweights were balanced (Table 5). The pullets weighed in weeks 3; 5; 8; 12, 13; 17 on average 181, 337, 678, 1,124, 1,432 g (EG 1); 178, 336, 671, 1,112, 1,450 g (EG 2) and 178, 337, 681, 1,116, 1,441 g (EG 3). The bodyweight development was even in all groups throughout the whole RP and neither the provision of environmental enrichment nor the reduction of stocking density had a statistically significant effect (Figures 1B and 2D). However, in week 17, with the reduced stocking density, the bodyweight improved slightly but not significantly. That means, in this week of life the pullets in EG 2 had a slightly higher bodyweight than in EG 1 and EG 3, but this difference was not significant (Figure 1B). The bodyweight declined significantly with increasing dust concentration of PM10 (regression coefficient: –21.64; 95% CI for the difference: –37.44 to –6.15) and increased significantly with every week of age (regression coefficient: 95.76; 95% CI for the difference: 93.03 to 98.57) (Figure 2D). In week 17, the decrease of the bodyweight due to increased dust concentration was significant as well (regression coefficient: –13.33; 95% CI for the difference: –25.81 to –1.59).

**Table 5.** The results for bodyweight, difference to the target weight, and uniformity in the 3 experimental groups (EG) in each assessed week of life (wk).

Response	EG	wk	EE	P./m²	n	Mean	SD	Min.	Max.
	1	3	no	>20	6	181	11	168	193
	2	3	yes	<20	6	178	11	164	191
	3	3	yes	>20	6	178	11	164	192
	1	5	no	>20	6	337	9	330	350
	2	5	yes	<20	6	336	9	324	346
	3	5	yes	>20	6	337	6	331	348
	1	8	no	>20	6	678	11	658	690
Bodyweight (g)									
	2	8	yes	<20	6	671	10	656	685
	3	8	yes	>20	6	681	16	664	711
	1	12, 13	no	>20	6	1124	60	1058	1190
	2	12, 13	yes	<20	6	1112	46	1053	1154
	3	12,13	yes	>20	6	1116	57	1056	1197
	1	17	no	>20	6	1432	23	1414	1470
	2	17	yes	<20	6	1450	21	1420	1477
	3	17	yes	>20	6	1441	28	1415	1487
	1	3	no	>20	6	−7.5	4.7	−13.1	−2.1
									
	2	3	yes	<20	6	−9.0	4.6	−14.8	−3.2
	3	3	yes	>20	6	−8.9	4.5	−14.9	−2.5
	1	5	no	>20	6	−7.6	2.1	−9.9	−4.7
	2	5	yes	<20	6	−8.0	2.1	−10.7	−5.8
	3	5	yes	>20	6	−7.7	1.4	−8.9	−5.3
Difference to the target weight (%)	1	8	no	>20	6	−2.5	1.7	−5.6	−0.4
	2	8	yes	<20	6	−3.5	1.6	−5.9	−1.2
	3	8	yes	>20	6	−2.0	2.3	−4.7	2.0
	1	12, 13	no	>20	6	4.0	2.1	1.4	6.6
	2	12, 13	yes	<20	6	2.9	1.0	1.0	3.6
	3	12,13	yes	>20	6	3.3	2.3	1.1	7.1
	1	17	no	>20	6	2.3	2.2	0.3	5.6
	2	17	yes	<20	6	3.6	1.3	2.1	5.2
	3	17	yes	>20	6	2.9	2.1	1.1	6.8
	1	3	no	>20	6	77.7	9.4	68.0	88.0
	2	3	yes	<20	6	72.3	7.5	60.0	82.0
	3	3	yes	>20	6	77.3	6.5	68.0	86.0
	1	5	no	>20	6	78.7	7.3	68.0	88.0
	2	5	yes	<20	6	73.7	5.7	66.0	82.0
	3	5	yes	>20	6	76.0	6.1	66.0	82.0
Uniformity (%)	1	8	no	>20	6	76.3	6.9	68.0	84.0
	2	8	yes	<20	6	72.0	6.7	62.0	78.0
	3	8	yes	>20	6	69.3	9.8	54.0	84.0
	1	12, 13	no	>20	6	77.0	6.0	70.0	86.0
	2	12, 13	yes	<20	6	83.3	4.7	78.0	88.0
	3	12,13	yes	>20	6	80.3	9.5	68.0	96.0
	1	17	no	>20	6	84.3	5.3	80.0	94.0
	2	17	yes	<20	6	81.7	8.7	66.0	92.0
	3	17	yes	>20	6	84.3	6.1	78.0	94.0

None of these differences were significant.

EE = environmental enrichment, P/m² = pullets per m², n = number of units included in the calculation, SD = standard deviation, Min. = smallest assessed value, Max. = largest assessed value.

The difference to the target weight was similar in all EG throughout all assessed weeks of life (Table 5). In weeks 3: 5; 8; 12, 13; 17 it was on average –7.5, –7.6, –2.5, 4.0, 2.3% (EG 1); –9.0, –8.0, –3.5, 2.9, 3.6% (EG 2) and –8.9, –7.7, –2.0, 3.3, 2.9% (EG 3). It was not significantly influenced by environmental enrichment or stocking density (Figures 1C and 2E). Throughout the RP, the difference to the target weight decreased significantly with increasing dust concentration (regression coefficient: –1.28; 95% CI for the difference: –2.11 to –0.49) and improved with increasing age (regression coefficient: 1.07; 95% CI for the difference: 0.93 to 1.22). In week 17, the decreasing effect of dust concentration of PM10 was significant as well (regression coefficient: –0.96; 95% CI for the difference: –1.81 to –0.12).

Uniformity represents the percentage of animals meeting ±10% of the average bodyweight. In general, the uniformity was at minimum 54% and at maximum 96%. The uniformity was similar in all EG and none of the group differences were significant (Table 5; Figures 1D and 2F). The average uniformity in weeks 3; 5; 8; 12, 13; 17 was 77.7, 78.7, 76.3, 77.0, 84.3% (EG 1); 72.3 73.7, 72.0, 83.3, 81.7% (EG 2) and 77.3, 76.0, 69.3, 80.3, 84.3% (EG 3). With increasing dust PM10 concentration, the uniformity decreased significantly (regression coefficient: –2.68; 95% CI for the difference: –4.67 to –0.64) and with every week of age it improved significantly (regression coefficient: 0.89; 95% CI for the difference: 0.52 to 1.27).

### Environmental Enrichment and Litter

The enrichment was used intensively right from the beginning. After 3 wk, 27% of the pecking blocks and 39% of the pecking stones were used up. In RP 1, 100% of the blocks and 90% of the stones were consumed after 8 wk. In RP 2, 76% of the blocks and 100% of the stones were used up in the same period. The consumption of the lucerne bales was not documented. They were replaced 1 to 2 times during each RP.

Litter quality of score 5 could already be seen in both RP by the first assessment visit after opening the cages and giving the pullets access to the litter area (A3, week 8). The litter crust covered on average 29.3% of the litter area in the units of EG 1, 24.0% in the units of EG 2, and 35.9% in the units of EG 3. On top of the crust was about 2 cm of loose litter (quality score 2). In RP 1, the quality of the litter was mainly of score 2, whereas in RP 2, half of the measurements had score 2 and the other half score 3. The average depth of the litter was about 5 cm after 8 wk and about 10 cm during the last weeks of the RP.

### Microclimate

The results of the microclimate measurements can be seen in Table 6. Light intensity in the cage levels ranged from 13 to 28 lx. Two-thirds of the average values measured were below 20 lx. The reduction of the light intensity was used as a management measure to reduce the number of cannibalistic injuries. The data were similar in both RP but differed between the units.

**Table 6.** The average values of the microclimate measurements in each unit and rearing period represented as mean values and ranges (in parentheses) for each experimental group (EG), with the exception of the largest value for ammonia (ppm), which represents the absolute highest value that was measured in this EG.

EG	1		2		3	
Rearing period	1	2	1	2	1	2
Light intensity (lx)	20 (13–28)	18 (14–23)	18 (13–24)	18 (14–22)	19 (15–24)	16 (14–18)
Ammonia (ppm):						
Mean value	0.0	5.9 (2.2–9.7)	0.0	3.6 (3.1–4.4)	0.0	5.5 (0.5–8.1)
Largest value	0	25	0	10	0	19
Temperature (°C)	25.0 (24.1–25.5)	19.6 (17.0–21.6)	24.9 (24.7–25.3)	18.9 (18.5–19.1)	24.7 (23.3–25.7)	18.6 (14.7–20.9)
Humidity (%)	59.7 (57.7–61.4)	51.8 (45.5–57.3)	61.2 (58.5–65.8)	52.1 (46.2–61.7)	60.6 (59.4–61. 9)	53.2 (47.6–58.6)
Dust PM10 (mg/m²)	4.66 (1.81–6.35)	6.46 (3.12–10.44)	5.78 (3.42–7.24)	5.73 (4.78–6.44)	4.10 (0.64–6.22)	5.54 (1.66–8.07)

PM10 = particulate matter 10 (particles <10 μm).

The measurements of ammonia in RP 1 were <5 ppm. During RP 2, most of the measured values were below 10 ppm; only during the last 2 assessment visits did a few values exceed 10 ppm with a maximum of 25 ppm measured in unit 3 at the last assessment.

The temperature measured and compared between the 2 RP differed widely. The mean temperature in RP 1 was 24.9°C and in RP 2 it was 19.0°C (Table 6).

The humidity differed substantially between the units and when comparing the RP. During RP 1, the humidity was between 57.7% (unit 8) and 65.8% (unit 1) and was higher than during RP 2 with a range from 45.5% (unit 8) to 61.7% (unit 1) (Table 6).

The dust concentration measurements varied greatly during each RP but did not differ as much when comparing RP 1 and RP 2 with each other. A decrease in dust concentration was measured from unit 1 to unit 9 in both RP. In general, units 1 to 6 had a higher dust concentration than units 7 to 9. The total dust concentration was on average between 11.53 and 23.08 mg/m³ in units 1 to 6 and between 1.34 and 12.40 mg/m³ in units 7 to 9. The data showed a very strong correlation (*r* = 0.99) between PM2.5 and PM10, which makes it impossible to estimate the influence of both values simultaneously on the response variables. For further evaluation, this study will focus on the PM10 concentration. The concentration of PM10 was between 5.43 and 10.44 mg/m³ in units 1 to 6 and between 0.64 and 4.78 mg/m³ in units 7 to 9 (Table 6).

## DISCUSSION

### Plumage Condition

Even though the pullets of EG 2 and EG 3 had access to the environmental enrichment from day 1 and used it intensely from the beginning, its positive effect on the plumage triscore was only significant in week 17. Other studies showed a distinct and significant effect of enrichment on the plumage condition. Feather pecking can be prevented almost entirely during the RP, as described by McAdie et al. (2005), if the pullets have access to enrichment from the 1st day of age. Feather pecking increased when environmental enrichment was provided for the first time when the pullets were 22- or 52-days-old, and it was most prevalent when enrichment was not provided at all (McAdie et al., 2005). Furthermore, feather pecking can be prevented effectively during rearing if the pullets have the option of pecking and scratching on the ground (Blokhuis and Van Der Haar, 1989) and their foraging behavior is encouraged (Huber-Eicher and Wechsler, 1998). A possible explanation for our finding of similar plumage triscores in all 3 EG and a non-significant effect of enrichment on the feather condition, except for week 17 when its effect was significant, could be that litter was first provided at 5 wk of age in all 3 EG. Johnsen et al. (1998) emphasized the importance of litter provision in the first 4 wk of age for preventing feather pecking: chicks reared on wire in the first 4 wk of age showed significantly more severe feather pecking at 5 to 6 wk of age compared with animals that were reared on straw in the first 4 wk. Furthermore, Johnsen et al. (1998) observed that feather pecking could not be prevented even after the provision of litter in week 5 once it had started. Thus, in the present study, the missing litter in the first weeks of life could have caused feather pecking in all 3 EG. Helmer (2017) and Zepp et al. (2018), who performed the behavioral observations on the same pullets that we assessed during the cage and the aviary phase in our project, confirmed that feather pecking occurred from the beginning and in all EG.

The plumage triscore of the pullets reared with a density of <20 pullets per m² was not significantly better than that of the pullets reared with a higher stocking density. Other studies came to the conclusion that keeping pullets at a high stocking density causes a significant increase in feather pecking and an inferior plumage quality during the RP (Wells, 1972; Hansen and Braastad, 1994; Huber-Eicher and Audigé, 1999; Bestman et al., 2009). Hens that already showed signs of feather pecking during rearing have a high likelihood of also performing this behavior during laying, independently of the later housing system (Johnsen et al., 1998; Bestman et al., 2009; Lambton et al., 2010; Gilani et al., 2013). In our study, the reason why the reduced stocking density had no significant decreasing effect on the occurrence of plumage damage might be that the stocking densities we examined were possibly too high in general and the differences too small. Other studies that investigated the influence of stocking density on the plumage quality during the RP used lower densities than we did and found that pullets kept at <10 birds per m² showed significantly less feather pecking and feather damage compared with pullets kept at higher densities (Wells, 1972; Hansen and Braastad, 1994; Huber-Eicher and Audigé, 1999). Huber-Eicher and Audigé (1999) studied the occurrence of feather pecking in 64 flocks on different commercial rearing farms and found that flocks kept at ≥10 pullets per m² had a 6.4 times higher risk of being affected by feather pecking than pullets kept at <10 birds per m². The authors suggested that 10 pullets per m² could be the biological threshold between high and low stocking density.

In our study, the most noticeable difference in the plumage triscore seemed to be between the EG with environmental enrichment and a lower stocking density (EG 2, average score: 10.61) and the EG without enrichment and with a higher density (EG 1, average score: 10.40). However, we found no statistically significant effect of enrichment or reduced stocking density on the plumage condition. Zepp et al. (2018), who conducted behavioral observations during the aviary phase in this experiment, found that the pullets in EG 1 showed significantly more gentle and severe feather pecking than the pullets in EG 2 and EG 3, and those in EG 3 (with environmental enrichment and with a higher stocking density) showed significantly more severe feather pecking than those in EG 2. In addition, Zepp et al. (2018) found a significant deteriorating effect of severe feather pecking on the plumage condition. It therefore seems that the plumage score system we used, even though single damaged feathers were counted on the body regions, was not precise enough to reflect these significant behavioral differences.

In both RP, two-thirds of the measured values of light intensity in the cages were below the recommended 20 lx (Lower Saxony State Office of Consumer Protection and Food Safety, 2013). Kjær and Vestergaard (1999) observed that laying hen pullets showed significantly more gentle feather pecking when the light intensity was low (3 lx) and 2 to 3 times higher rates of severe feather pecking when the light intensity was high (30 lx). As a result, the plumage condition was poorer at 30 lx than at 3 lx during the RP (Kjær and Vestergaard, 1999). Other authors also found a relationship between high light intensity during rearing or at the beginning of lay and the occurrence of feather pecking in pullets or young laying hens (Hughes and Duncan, 1972; Drake et al., 2010). In our study, the light intensity did not have an effect on the plumage quality.

Concerning the microclimate, the recommended temperature by Lohmann Tierzucht GmbH (2017) of 18 to 20°C from 5 wk of age could be maintained during RP 2 with an average temperature of 19.0°C in the housing system. During RP 1, the average temperature was 24.9°C. It was warmer because RP 1 took place in summer whereas RP 2 took place in winter. For pullets older than 4 wk of age, such high temperatures in the housing system during summer should be prevented with sufficient ventilation. Heat compromises the physiological thermoregulation, and heat stress results in immunosuppression and reduced production performance (amongst other consequences) in laying hens and broilers (Bartlett and Smith, 2003; Mashaly et al., 2004). The plumage triscore in RP 1 was lower than during RP 2. The regression model confirms the correlation between increased temperature and poorer plumage quality. Lambton et al. (2010) also observed that a prolonged warm climate leads to stress and a decrease in plumage condition in laying hens. In contrast, Green et al. (2000) documented that a temperature below 20°C in the housing system results in a higher risk of feather pecking.

During both RP, the plumage triscore decreased over time in all 3 EG (on average –0.21 score points per week). The plumage quality decreased relatively evenly during RP 2, whereas the decrease in RP 1 peaked around week 8 to week 12 and proceeded afterwards with a slight further decrease or even turned into an improvement of the plumage. A possible explanation for this development in RP 1 could be molt, but a more likely one is the high temperature in the housing system during this time. The average temperature documented during this time was 24.8°C (A3) and 32.0°C (A4). When the accelerated decrease in plumage quality had stopped (A5), 19.8°C was measured. Wechsler et al. (1998) described a sudden and strong increase of feather pecking in week 4. Johnsen et al. (1998) observed that feather pecking peaked around the 7th week of age, but at the end of the RP with 18 wk, all birds showed an intact plumage. The author assumed this change was due to molt.

### Skin Injuries

91% of the observed injuries had a size of ≤0.5 cm, which we interpret as a sign of pecking damage. Furthermore, the mainly injured body regions were the tail and the back, regions which are also affected by feather pecking (Zepp et al., 2018). Therefore, we assume the documented skin injuries are the result of cannibalistic pecking.

The 2 EG reared with environmental enrichment showed slightly less injured body regions (–0.02 injured body regions) than the EG without enrichment, but the statistical analysis could not prove a significant effect of environmental enrichment on body injuries. Huber-Eicher and Wechsler (1998) and Johnsen et al. (1998) noted that pullets reared with enrichment and provided with foraging materials showed fewer cannibalistic injuries during the RP than pullets without these provisions. The same effect was documented on young turkeys (Martrenchar et al., 2001). Analog to the effect on feather pecking, the missing litter in the first 4 wk of life probably increased the number of body injuries in our study. Pullets reared on wire instead of litter in the first 4 wk showed significantly more cannibalistic injuries during rearing than pullets reared on litter (Johnsen et al., 1998).

There are different opinions on whether a relationship exists between feather pecking and the incidence of cannibalistic injuries. Gunnarsson (1999) found no significant correlations between both behavioral disorders. Kjær and Vestergaard (1999) described that severe feather pecking could possibly develop into vent cannibalism. Other authors observed that the same housing conditions that result in an increase of feather pecking also result in an increase of cannibalistic injuries (Allen and Perry, 1975; Huber-Eicher and Wechsler, 1998) even though these 2 response variables originate from distinct behavioral patterns (Allen and Perry, 1975). The results of our study confirm these observations by showing a similar distribution of both plumage damage and body injuries in the different EG.

Other authors could not detect a significant effect of stocking density on damaging cannibalistic pecking in pullets and young laying hens (Hughes and Duncan, 1972). The statistical analysis in our study also did not show a significant effect.

A high light intensity during the RP can increase the occurrence of damaging pecking (Hughes and Duncan, 1972). Kjær and Vestergaard (1999) observed that a higher light intensity (30 vs. 3 lx) during rearing caused an increase in cannibalism during the laying but not during the RP. This is in accordance with our findings that light intensity does not have an effect on skin injuries during rearing.

In RP 2, during which the recommended temperature of 18 to 20°C for pullets older than 4 wk of age (Lohmann Tierzucht GmbH, 2017) could be maintained, the pullets showed fewer body injuries per bird than in RP 1 with an average temperature of 24.9°C. The regression model showed a positive relationship between temperature increase and the number of injured body regions.

The occurrence of body injuries peaked around week 8 and week 12 in RP 2 and RP 1, respectively, and stabilized afterwards at a lower level. Johnsen et al. (1998) observed the same development but documented most of the injuries between the 4th and 7th week of age. Despite the mentioned occurrence of injuries on the body regions, we emphasize that the number of skin injuries in our study was altogether very low.

### Head Injuries

The provision of enrichment had no significant effect on head injuries. In contrast, in adult laying hens, the access to environmental enrichment reduced the number of aggressive head pecks significantly (Gvaryahu et al., 1994; Jones et al., 2002). Jones et al. (2002) concluded that pecking at the enrichment devices prevents the hens from potentially injurious pecking at other birds. Regarding the stocking density, our statistical analysis could not find a significant effect on the number of head injuries. Some studies came to the same conclusion that aggressive head pecking was unaffected by the stocking density in laying hens (Cunningham and Gvaryahu, 1987; Carmichael et al., 1999). However, Ali and Cheng (1985) found that high stocking densities lead to an increase in comb damage.

### Bodyweight and Bodyweight Uniformity

In general, neither the provision of environmental enrichment nor the different stocking densities had a significant effect on the bodyweight of the pullets. Obviously, the environmental enrichment devices did not serve as substitution for chicken feed. The pullets reared with enrichment must have consumed sufficient feed as the balanced bodyweights indicate. Our results support previous findings that stocking density has no significant effect on bodyweight in laying hens (Steenfeldt and Nielsen, 2015; Widowski et al., 2017). However, other studies found that a higher stocking density leads to a significantly lower bodyweight during rearing or during the laying period (Wells, 1972; Carey, 1987; Cunningham and Gvaryahu, 1987; Onbaşılar and Aksoy, 2005; Sarica et al., 2008). In our study, this effect seemed only to be true in week 17 when the reduced stocking density had a slight positive influence on the bodyweight. But this was not significant. A higher stocking density can be associated with a poorer food intake in laying hen pullets and adults and in other poultry (Carey, 1987; Cunningham and Gvaryahu, 1987; Thomas et al., 2004; Dozier et al., 2006; Nahashon et al., 2006). The mentioned studies came to different conclusions independent of the used ranges of stocking densities. Comparing the feeder space per bird and the daily feeding frequency with other studies does not give a conclusive indication on why the mentioned studies showed different results (Wells, 1972; Carey, 1987; Cunningham and Gvaryahu, 1987; Onbaşılar and Aksoy, 2005; Sarica et al., 2008; Steenfeldt and Nielsen, 2015; Widowski et al., 2017). Thus, there must have been other factors involved.

In our study, Unit 1 was the nearest to the ventilation fans and as a result the first units had a higher dust concentration as the air from the barn was sucked into their direction and into the ventilation fans. We found a significant correlation between rising dust concentration and the decrease of bodyweight. Willis et al. (1987) observed, in accordance with our results, that broilers reared in an environment with reduced dust concentration gained more weight than broilers reared with the prevalent dust concentration.

The provision of environmental enrichment or the reduced stocking density did not seem to have an impact on bodyweight uniformity. In the literature, different results on the effect of reduced stocking density on bodyweight uniformity can be found. Wells (1972), in accordance with our findings, could not detect significant differences in the bodyweight variation of the pullets between different stocking densities (5.4, 7.2, 10.8, 14.3 birds per m²). Widowski et al. (2017) did not observe an impact in laying hens (14.3 compared with 19.2 birds per m²). In contrast, Petek et al. (2010) found that the uniformity of broilers was significantly reduced at the highest stocking density (23 birds per m² compared with 15 and 19 birds per m²). The choice of stocking densities does not seem to be the reason for the contrasting results of the last-mentioned study. Instead, broilers that gain a lot of weight in a short period may be more sensitive to housing factors in terms of bodyweight gain compared with laying hens.

During the RP, the bodyweight uniformity of the pullets improved significantly with increasing age. According to Lohmann Tierzucht GmbH (2017), an average uniformity of 76.6 to 78.8% is moderate. The recommended value of at least 80% in week 15 to week 16, when uniformity is supposed to be at its highest level during rearing, was achieved in week 12, 13 (EG 2 and EG 3) and in week 17 in all 3 EG.

## CONCLUSION

In our study, the positive impact of environmental enrichment was only significant in week 17 on the plumage condition, whereas the reduced stocking density never had a statistically significant effect on the occurrence of feather pecking or skin injuries. However, we found reduced stocking density to have a slight positive effect on the plumage condition and enrichment to slightly reduce the number of injured body regions. A significant increase in plumage damage and in the number of injured body regions occurred in connection with increasing temperature in the housing system and increasing age of the pullets. Such welfare problems can be reduced with sufficient ventilation. Furthermore, in our study most of the injuries occurred in the 8th or 12th week of life. In this period, pullets should be supervised as a preventive measure. Due to the significant positive effect that environmental enrichment had on the prevention of feather damage in week 17 and its slight positive influence on skin injuries, its effect should be discussed. It remains to be evaluated if the impact of this kind of enrichment is in general limited. It is possible that the negative effect of missing litter at the beginning of the RP, resulting in an unsatisfied foraging and pecking behavior, outweighed any potentially positive effect of the provided enrichment devices. Additionally, a more detailed assessment system seems to be needed to identify the differences in the feather damage between the EG. The plumage score system we used was not able to reflect the significant differences in the damaging pecking behavior of the pullets in the 3 EG (Zepp et al., 2018). Possibly, the 2 stocking densities we used were too high to show a significant positive effect of reduced stocking density on animal welfare. The impact of densities lower than those we used, including values of <10 and >10 pullets per m², should be further researched. In summary, our study showed the importance of management arrangements adjusted to the pullets’ age and concerning temperature in the housing system to ensure animal welfare of pullets. The influence of environmental enrichment and the reduction of the stocking density need to be discussed and further researched to clearly identify their impact.
